# Lactation Support as a Proxy Measure of Family-Centered Care Quality in Neonates with Life-Limiting Conditions—A Comparative Study

**DOI:** 10.3390/children10101635

**Published:** 2023-09-30

**Authors:** Suneeta Brito, Allison Williams, Jenny Fox, Tazuddin Mohammed, Nayef Chahin, Kaitlin McCarthy, Lamisa Nubayaat, Shirley Nunlist, Mason Brannon, Jie Xu, Karen D. Hendricks-Muñoz

**Affiliations:** 1 Division of Neonatal Medicine, Department of Pediatrics, Children’s Hospital of Richmond at VCU, Virginia Commonwealth University School of Medicine, P.O. Box 980646, Richmond, VA 23298-0646, USA; suneeta.brito@vcuhealth.org (S.B.); jenny.fox@vcuhealth.org (J.F.); tazuddin.mohammed@vcuhealth.org (T.M.); nayef.chahin@vcuhealth.org (N.C.); jie.xu@vcuhealth.org (J.X.); 2 Department of Psychology, Virginia Commonwealth University School of Medicine, P.O. Box 980646, Richmond, VA 23298-0646, USA; allison.williams@vcuhealth.org; 3 Eastern Virginia School of Medicine, P.O. Box 1980, Norfolk, VA 23501-1980, USA; mccartk@evms.edu; 4 Cornell University, P.O. Box 752, Ithaca, NY 13853, USA; ln234@cornell.edu; 5 Virginia Commonwealth University School of Medicine, P.O. Box 980646, Richmond, VA 23298-0646, USAmason.brannon1@vcuhealth.org (M.B.)

**Keywords:** life-limiting conditions (LLCs), neonatal intensive care unit (NICU), family-centered care, lactation, breastfeeding, kangaroo mother care (KMC)

## Abstract

Background: Lactation support is an important measure of Family-Centered Care (FCC) in the Neonatal Intensive Care Unit (NICU). Life-limiting conditions (LLCs) raise complex ethical care issues for providers and parents in the NICU and represent a key and often overlooked population for whom FCC is particularly important. We investigated healthcare disparities in FCC lactation support quality in infants with LLCs. Methods: A retrospective cohort of inborn infants with or without LLCs admitted to the NICU between 2015–2023 included 395 infants with 219 LLC infants and 176 matched non-LLC infants and were compared on LLC supports. Results: The LLC cohort experienced greater skin-to-skin support, but less lactation specialist visits, breast pumps provided, and human milk oral care use. LLC infants also experienced less maternal visitation, use of donor milk (LLC: 15.5%, non-LLC: 33.5%), and breastfeeds (LLC: 24.2%, non-LLC: 43.2%), with lower mean human milk provision (LLC: 36.6%, non-LLC: 67.1%). LLC infants who survived to discharge had similar human milk use as non-LLC infants (LLC: 49.8%, non-LLC: 50.6%). Conclusion: Lactation support was significantly absent for families and infants who presented with LLCs in the NICU, suggesting that policies can be altered to increase lactation support FCC quality for this population.

## 1. Introduction

Family-Centered Care (FCC) strives to improve infant health and maternal outcomes through parental empowerment and partnership with medical providers. FCC encompasses four basic values: dignity and respect, information sharing, family participation in infant care and family collaboration [1]. Utilization of FCC in the NICU and specific FCC practices such as Kangaroo Mother Care (KMC) and breastfeeding have been beneficial in reducing infant length of stay, with improved neurobehavioral outcomes in neonates and optimized growth [2,3,4]. Parents provided this opportunity also experience reduced parental stress and improved parent–infant bonding with greater breastmilk utilization before discharge and readiness for discharge [1,5,6,7,8,9]. The NICU is a stressful environment, with high risk for parent–infant separation that can influence and interrupt important mother–infant attachment opportunities important to healing and FCC. For infants that present with life-limiting conditions the NICU can be especially stressful.

Pediatric life-limiting conditions (LLCs) are defined as conditions for which there is no reasonable hope of cure and for which progression of the disease is fatal with increased risk of death before reaching adulthood. Over the past several years, with advances in medical therapies, an increasing majority of parents of infants with LLCs have advocated for life-sustaining therapies despite life-limiting diagnoses contributing to a higher incidence of LLC children requiring intensive medical care in the NICU. Recent studies identified that, while 18% of neonates had life-limiting or life-threatening conditions, only 1.5% of parents elected to solely provide comfort care and forego life sustaining therapies [5]. Furthermore, in 2012, Johnson et al. identified that termination of pregnancy (TOP) after a prenatal diagnosis was 83% for anencephaly fetuses and 63% for fetuses with spina bifida [10]. TOP for spina bifida was only more common with prenatal diagnosis at less than 24 weeks gestation and with defects of greater severity. Of note, geographical differences identified greater pregnancy termination in Europe as compared to North America in their population [10]. An additional study by Janvier et al. in 2012 identified that, in children diagnosed with trisomy 13 and 18, 30% of parents chose “full” interventions with 30% of these children surviving to a median age of 4 years [11]. Importantly, despite the low survival rates and severe disabilities, 97% of parents described their child with trisomy 13 or 18 as happy, with parental reports that these children enriched their family irrespective of longevity [11]. Thus, the changing and growing NICU LLC complement presents challenges to optimize alignment of NICU provider perceptions of care provision and utilization of NICU resources with parent care decisions for the mother–infant dyad with a neonatal life-limiting condition [12].

FCC policies and algorithms in the NICU are developed and implemented to enhance FCC practices of skin-to-skin, breastfeeding, provision of the mother’s own milk, or use of donor human milk based on NICU provider perception of treatment value within the context of the infant’s medical condition with a goal of optimal resource utilization. These policies may be further complicated by the needs of the mother–infant dyad where the infant presents with life-limiting conditions. As such, the aim of this study was to assess the quality of FCC to optimize our care of this growing and critical population. To address this aim, we identified breastfeeding outcomes in LLC infants compared to non-LLC infants, examined differences in provider support, and determined maternal engagement between LLC and non-LLC infants to identify barriers in the provision of FCC lactation engagement.

## 2. Materials and Methods

### 2.1. Design

Using a semi-matched control design, the study is a retrospective chart review from January 2015 to December 2022 of infants admitted to the Children’s Hospital of Richmond (ChoR) at the Virginia Commonwealth University (VCU), a Level IV urban regional referral NICU with capabilities to provide the highest level of obstetrical and neonatal critical care. The study evaluated breastfeeding outcomes in LLC infants compared to non-LLC infants and examined differences in provider support and maternal engagement between the LLC and non-LLC groups with examination of other known barriers to lactation engagement such as maternal race, ethnicity, or non-English language for differences in contribution of lactation support. The research study was approved as exempt by the Virginia Commonwealth University Institutional Review Board (IRB).

As a NICU standard of care, all infants were afforded private rooms with rooming-in and sleeping facilities for the parents. Families were provided FCC services as a standard of care that included occupational/speech feeding therapy, lactation consultations, religious support, psychological services, and physical therapy.

### 2.2. Study Population

The LLC cohort included neonates with lethal or nonlethal life-limiting conditions who survived at least one week, including those with congenital malformations and moderate to severe Hypoxic Ischemic Encephalopathy (HIE) [13]. HIE was included as many infants who have suffered moderate to severe HIE have increased mortality in the newborn period with greater risk for medical conditions including cerebral palsy, severe motor and cognitive impairment, feeding intolerance and nutritional difficulties, learning disabilities, sensory impairment, and seizures that are life-limiting [14]. The non-LLC cohort was matched by gestational age (to address potential differences in developmental supports by gestational age as well as differences in milk production by gestational age), birth year (to address potential differences in lactation support offered by year of NICU admission), and maternal race/ethnicity. Infants were identified by diagnostic codes using the NICU Vermont Oxford Network database from 2015 to 2022 for full medical record extraction. Study exclusion criteria included lactation-limiting factors (maternal HIV positivity and mothers with mastectomies), infant survival of less than 1 week, and infant transfer into the CHoR NICU after the first week of life.

### 2.3. Data Inclusion and Analysis

Maternal demographics included age, race/ethnicity, primary language, zip code, and employment and insurance status (Medicaid, private or uninsured) as a proxy for income. Maternal health data collected included length of antepartum stay, mode of delivery, severe maternal complications (blood transfusion, intensive care admission), maternal substance use, and maternal mental health history. Maternal engagement included the number of maternal visits, as well as first time and number of times maternal skin-to-skin contact was performed.

Infant health characteristics collected included singleton status, diagnosis, prenatal identification of LLC, gender, gestational age in weeks, infant complications (surgeries, extracorporeal membrane oxygenation (ECMO) during first week), hospital length of stay, do not resuscitate (DNR) status, and discharge status (home, transitional care, death). FCC details included resource supports offered prenatally or during the infant’s first week of life. Prenatal support included discussion of maternal breastfeeding preferences. NICU feeding preferences included documentation of breastfeeding/pumping preferences during the first week of life and day of life, when a breast pump was offered, early lactation support, provision of infant oral care with mother’s milk, lactation consultation, occupational therapy, and physical therapy consultation. Infant nutrition outcomes included percentage of breastmilk provided at discharge, date of first Pasteurized Donor Human Milk (PDHM) offering, and type of nutrition utilized during the first week of life.

### 2.4. Data Analysis

Data was analyzed using IBM SPSS Statistics. Pearson correlations, *t*-tests, Chi-squared, and ANOVAs were conducted to examine lactation outcome and lactation support differences between the LLC and the non-LLC control cohorts. Within-group analyses were conducted using three ANOVAs with post hoc planned contrasts for maternal income, maternal race, and maternal primary language.

## 3. Results

### 3.1. Patient Population

The study population included 395 infants who met the inclusion criteria, of which 219 infants were included in the LLC cohort and 176 in the non-LLC cohort. There were no differences in the sociodemographic conditions, race, or ethnicity among the populations; see Table 1. Infant characteristics of the two cohorts, including types of LLC, lethal, and non-lethal conditions, are displayed in Table 2.

### 3.2. Influence of Infant LLC Diagnosis on Access to Early Provider Lactation Support and Maternal Breastfeeding Preference

In the assessment of early provider lactation support for the LLC infants, there were no differences noted in the rate of consultations for physical (PT) and speech therapy services compared to the non-LLC group, as shown in Table 3. There was a significantly greater provision of skin-to-skin education for mothers with LLC compared to non-LLC infants, *p* < 0.001, but a significantly decreased utilization of formal lactation consultations in the LLC, *p* < 0.05, compared to the non-LLC group, as shown in Table 3.

Investigating the impact of LLC diagnosis on breastfeeding preference, mothers of the LLC group were less likely to express a prenatal preference for provision of breastmilk with 44% of LLC mothers expressing prenatal preference of breastfeeding compared to 63% of non-LLC mothers, *p* < 0.001, as shown in Table 3. In evaluating maternal preference for breastfeeding after delivery in the NICU, of the mothers queried, neither LLC mothers nor mothers of non-LLC infants varied in their postnatal breastmilk preferences in the NICU, Table 3. Additionally, the rates of provision of breast pumps to assist in lactation for milk production support was significantly less likely to be provided in the LLC maternal cohort compared to the non-LLC mothers, with 46% of LLC mothers provided this resource compared to 58% of non-LLC mothers, *p* < 0.001, as shown Table 3.

### 3.3. Influence of Infant LLC Diagnosis on Early Maternal Engagement during the First Week of Life

Maternal visitation was significantly less among LLC infants during the first week of life compared to maternal visitation among non- LLC infants, *p* < 0.006, as shown in Table 4. In evaluating maternal provision of skin-to-skin in infants with LLC, mothers engaged in skin-to-skin care to a greater degree than mothers with non-LLC infants during the first week of life, *p* < 0.001, as shown in Table 4. However, in the provision of the mother’s own milk for infant oral care, LLC infants were less likely to receive this level of care compared to non-LLC infants, *p* < 0.001, as shown in Table 4.

### 3.4. Influence of Infant LLC Diagnosis on Breastfeeding Outcomes

**Figure 1:**
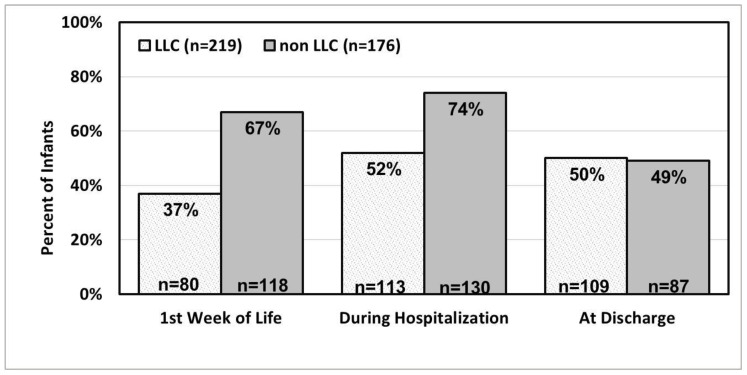
The utilization of percent of breastmilk during the first week, during hospitalization, and at discharge in LLC and non-LLC infants. *n* = 395.

In evaluating the impact of the life-limiting condition on the use of breastmilk for their infants at various times of the infant’s hospitalization and at infant discharge, there were a significantly greater number of infants in the non-LLC group who received a higher percentage of breastmilk during the first week of life and during their hospitalization compared to the LLC group, *p* < 0.001, as shown in Figure 1.

When an infant’s provision of breastmilk at discharge was evaluated, there was no statistical significance between the percentage of breastmilk at discharge for LLC infants (*n* = 109/219, 50%) compared to non-LLC infants (*n* = 87/176, 49%), as shown in Figure 1.

### 3.5. Influence of Maternal Race, Ethnicity, and the Non-English Language on Infant LLC Lactation Supports

In the assessment of the influence of maternal race and ethnicity on the availability and provision of lactation support among the LLC cohort of infants and their mothers, we found no differences in the provision of donor milk, breast pumps, or provider supports by maternal race, ethnicity, or maternal socioeconomic characteristics such as income. More specifically, assessment of maternal income, maternal race/ethnicity, and percentage of breastmilk at discharge was not different among the LLC and non-LLC group, F (2,202) = 1.1, *p* = 0.324.

In assessment of the influence of maternal language on breastfeeding at discharge there was a notable difference in the rate of provision of breastmilk at discharge, F (2,209) = 3.84, *p* < 0.01. Specifically, English-speaking mothers’ infants experienced higher rates of breastmilk at discharge compared to Spanish-speaking mothers’ infants *p* < 0.05, 95% CI = 0.10, 1.52).

## 4. Discussion

Family-centered care is a philosophy of care practice known to elevate care quality and outcome for parents and infants who are admitted to the NICU, with provider and parent communication and shared decision making a hallmark of FCC. The present study examined FCC lactation support and outcomes in infants with life-limiting conditions as a proxy for FCC quality, to highlight and attain greater understanding of the availability of FCC and use of this resource for this growing critical population in the NICU. Parents with infants with LLCs require critical communication that includes parental care goals for their infant through shared decision making. FCC practices such as provision of the mother’s own milk and skin-to-skin care that contribute a pivotal role in mitigating many adverse consequences and challenges for infants are early key discussion goals to address hospital resources including lactation support education or use of donor milk sources as a bridge to the mother’s own milk.

This study identified significant disparities in utilization of human milk and breastmilk resources at discharge for LLC compared to non-LLC infants. Hospital resources of lactation specialists and breast pumps offered to mothers were significantly lower in the LLC group. We speculate that this could be attributed to LLC mothers’ uncertainty of the survivability of the infant as well as provider and parental perception related to the infant’s potential longevity or possible improvement as well as communication of the practice of infant comfort care. Of interest, there was significantly greater early provider engagement in education of mothers in skin-to-skin care for infants with LLC including early consultation with occupational therapists for maternal provision of this training. One speculation related to this finding is that provider perception of advancing skin-to-skin care, infant bonding, or touch may be associated with minimizing parental grief and psychological pain, thereby leading to a provider’s parental-protective recommendation to advance this practice to alleviate parental stress and attachment trauma in infants with an LLC diagnosis. An additional finding that lactation-specific oral care, lactation specialist consultation, and offering of human donor milk was also decreased for LLC infants may align with provider perception of economic-resource-limited utilization for an infant with a shortened life expectancy. These results identified that, while maternal- and occupational-therapist-provided skin-to-skin contact in the LLC group superseded the non-LLC group, in general, the LLC infant’s mother had limited lactation support and the infant had limited initial human milk provision. The findings related to decreased maternal visitation in the first week of life in the NICU may reflect the complications of maternal illness with mothers recovering from their own conditions and logistic issues with maternal or infant transfers and NICU visitation limitation that impacted maternal infant separation policies for the time period of COVID-19. Nevertheless, mothers with LLC had relatively more skin-to-skin exposure compared to non-LLC mothers, indicating an early support that providers initiated for all infants. Furthermore, similar to previous findings, we identified non-English language as a barrier to breastmilk utilization in our cohorts [15,16]. Finally, contrary to previous studies demonstrating FCC and lactation healthcare disparities in the NICU for mothers of racial, ethnic, and socioeconomic differences, we did not identify race, ethnicity, or socioeconomic differences as a barrier to lactation support in the NICU in our cohort analysis [17,18,19,20,21,22,23,24].

The complex decision making for the infants with LLCs has significant consequences for patients and their families that require shared NICU healthcare team responsibility to optimize outcomes. Given the marked rise in the prevalence of infants in the NICU with a life-limiting or life-threatening condition as well as recent increased life expectancy for these infants, adjustments in NICU policies may be needed [25,26,27]. This could include communication of the often-expected presentation of a live born infant linked with the limitations of the infant’s diagnosis. These communications will be of importance to guide NICU resource utilization as well as health team care practices reflective of respect for family care choices that may include intensive medical intervention to prolong life expectancy [25,26,27,28,29,30,31,32].

Our study had several study limitations inherent to design, sample, and instrumentation. First, data collected was cross-sectional over 8 years. During this time, in the era of COVID-19, we also transitioned between electronic medical record systems. Thus, data access limitations due to relocated or missing data may have impacted our findings. Additionally, skin-to-skin care was not documented for durations less than 60 min for all children, and donor milk was not available until 2017, with the initiation of a donor human milk program in collaboration with a regional milk bank. Finally, as a regional level IV referral NICU with maternal–fetal medicine specialists, as well as an international surgical program, approximately 25% of the population were infant transfers, with limited maternal demographics, prenatal consults, and pertinent medical information. Further limitations included a small sample size due to the complexity of the infants’ condition in the LLC group. Additionally, mixing of the mother’s own milk and donor milk prevented quantifying the type of human milk utilization amounts. While many of these limitations affected our cohorts equally, we feel that the focused adjusted matched cohort design aimed for addressed these limitations to providing important findings. The results expand the current knowledge of FCC practices and identify lactation engagement as a potential objective FCC quality proxy that can be used in the NICU to track FCC quality for unique clinical populations such as LLC infants and their families.

Our findings are relevant to other NICUs, as they identify potential resource and health team FFC challenges faced by the growing LLC population. The study also identified that all groups could benefit from increased support in lactation and skin-to-skin while providing us with opportunities to investigate and address barriers to lactation support as a quality indicator of FCC in our population of infants. Importantly, the sub-optimal utilization of human milk in the LLC group in early life improved over time and equaled the control group rate at discharge. Finally, while racial and ethnic health care disparities may occur in the NICU, within this study population and the LLC cohort we found no disparities in the provision of donor milk, breast pumps, or provider supports related to maternal race, ethnicity, or maternal socioeconomic differences. However, we did identify that infants of English-speaking mothers had higher rates of breastmilk at discharge compared to infants of Spanish-speaking mothers in the LLC and non-LLC groups, indicating a need to assess and improve current interpretation services within the NICU especially targeting the first weeks of admission to the NICU.

## 5. Conclusions

The NICU experience is extremely difficult and stressful for all parents, and support through culturally sensitive communication and trust throughout their infant’s stay is imperative. While challenging, the NICU environment for infants with unique diverse LLC diagnoses can still expect to provide optimal FCC practices including lactation support. Lactation support is one important measure of FCC quality in the neonatal intensive care unit. Human milk, either a mother’s own milk or donor milk, enhances quality of life for infants. Therefore, assessment of lactation support as a proxy for FCC provides one specific objective opportunity to address quality of the care delivered for health care teams to develop avenues to improve care for vulnerable children. In this study, lactation support services and lactation outcomes were used as a proxy for FCC quality in a growing NICU LLC population. Results identified that lactation support such as breast pumps, maternal lactation consult support, donor milk provision, and oral care were specifically limited for LLC infants. In our hands, the results identified that access to FCC practices of lactation supports were not associated with maternal racial or ethnic disparities but uncovered disparities among non-English-speaking families. Furthermore, the study identified that, overall, there was less-than-optimal lactation support during the first week of life for all infants regardless of diagnoses. Further research is warranted to understand provider and parenting perception as contributing factors to the identified lactation support disparities between non-LLC and LLC infants. Importantly, the results of our study provide first steps that may assist others in optimizing engagement protocols to enhance FCC with special consideration of lactation support for LLC infants and families.

## Figures and Tables

**Table 1:** Maternal demographics of the LLC and non-LLC study cohorts. *n* = 395.

Variable	LLC (*n* = 219)	Non-LLC (*n* = 176)	Total (*n* = 395)
Race *n* (%)			
Caucasian	82 (37)	73 (42)	155 (39)
African American	80 (37)	75 (43)	155 (39)
Asian/Pacific Islander	8 (4)	4 (2)	12 (3)
Other	20 (9)	4 (2)	24 (6)
Ethnicity *n* (%)			
Hispanic	32 (15)	28 (16)	60 (15)
Language *n* (%)			
English	174 (79)	154 (88)	328 (83)
Spanish	21 (10)	19 (11)	40 (10)
Mean Maternal Age ± SD (years)	28.3 ± 6.7	28.4 ± 5.8	28.3 ± 6.3
Insurance Type *n* (%)			
Public/Medicaid	94 (43)	104 (59)	198 (50)
Private	86 (39)	51 (29)	137 (35)
Uninsured	17 (8)	17 (10)	34 (9)
Employment Status *n* (%)			
Employed	54 (25)	81 (46)	135 (34)
Unemployed	56 (26)	64 (36)	120 (31)

**Table 2:** Infant characteristics of the LLC and non-LLC study cohort. *n* = 395.

Variable	LLC (*n* = 219)	Non-LLC (*n* = 176)	Total (*n* = 395)
Average gestational age ± SD (weeks) *	35.9 ± 3.9	35 ± 4.0	35.5 ± 4.0
Average length of stay ± SD (days) *	42.1 ± 47.9	28.8 ± 26.2	36.2 ± 40.2
Sex *n* (%)			
Male	121 (55)	110 (63)	231 (59)
Delivery type *n* (%)			
Vaginal	94 (43)	86 (49)	180 (46)
C section	125 (57)	90 (51)	215 (54)
Inborn N (%)	161 (74)	136 (77)	297 (75)
Surgeries in 1st week of life *n* (%)	75 (34)	7 (4)	82 (21)
ECMO in 1st week of life *n* (%)	6 (3)	0 (0)	6 (2)
Type of life-limiting condition N (%)			
Lethal neurological	27 (12.3)		
Lethal renal	10 (4.6)		
Lethal genetic	5 (2.3)		
Congenital heart	12 (5.5)		
Lethal gastrointestinal	4 (1.8)		
Lethal pulmonary	1 (0.5)		
Other lethal conditions	6 (2.7)		
LLC non-lethal	110 (50.2)		
Hypoxic Ischemic Encephalopathy	44 (20.1)		

ECMO = Extracorporeal membrane oxygenation. LLC non-lethal = Life-limiting conditions which are often life threatening but not lethal. Variables marked with * reflect *p* < 0.05 difference between LLC and non-LLC groups.

**Table 3:** Provider engagement, lactation support, and maternal breastfeeding preference.

	LLC (*n* = 219)	Non-LLC (*n* = 176)	Total (*n* = 395)
Mean ± SD (days that service was given)			
Physical therapy visits	0.57 ± 0.88	0.47 ± 0.67	0.52 ± 0.79
Occupational therapy skin-to-skin **	0.14 ± 0.42	0.01 ± 0.11	0.08 ± 0.32
Lactation consults *	0.33 ± 0.58	0.51 ± 0.72	0.41 ± 0.65
Speech therapy	0.28 ± 0.9	0.22 ± 0.68	0.26 ± 0.81
Breast pump provided *n* (%) **			
Yes	100 (46)	102 (58)	202 (51)
No	64 (29)	61 (35)	125 (32)
Breastmilk preference (prenatal) *n* (%) **			
Prefers breastfeeding	96 (44)	111 (63)	207 (52)
Refused breastfeeding	16 (7)	17 (10)	33 (8)
Consult but no BF preference	26 (12)	5 (3)	31 (8)
No consult	81 (37)	43 (24)	124 (31)
Breastmilk preference (NICU consult) *n* (%)			
Prefers breastfeeding	20 (9)	18 (10)	38 (10)
Refused breastfeeding	3 (1)	1 (0.6)	4 (1)
Consult but no BF preference	7 (3)	8 (5)	15 (4)
No consult	189 (86)	149 (87)	338 (86)

BF = breastfeeding (used interchangeably in this article with breastmilk), LLC = life-limiting condition, Non-LLC = non-life-limiting condition. Variables marked with * reflect *p* < 0.05 difference between LLC and control groups, variables marked with ** reflect *p* < 0.001.

**Table 4:** Maternal engagement in the 1st week of life in LLC and non-LLC infants. *n* = 395.

	LLC (*n* = 219)	Non-LLC (*n* = 176)	Total (*n* = 395)	*p* Value
Mean ± SD (days that service was given)				
Maternal visitation	2.63 ± 2.8	3.30 ± 1.8	2.92 ± 2.4	0.006
Maternal skin-to-skin	1 ± 2.1	0.30 ± 0.7	0.69 ± 1.7	<0.001
Oral care **	0 ± 0	0.29 ± 0.8	0.13 ± 0.6	<0.001

Variables marked with ** reflect *p* < 0.001.

## Data Availability

Data is unavailable due to institutional privacy restrictions.
